# Monitoring vigabatrin in head injury patients by cerebral microdialysis: obtaining pharmacokinetic measurements in a neurocritical care setting

**DOI:** 10.1111/bcp.12414

**Published:** 2014-10-20

**Authors:** Richard J Shannon, Ivan Timofeev, Jürgens Nortje, Peter J Hutchinson, Keri L H Carpenter

**Affiliations:** 1 Division of Neurosurgery, Department of Clinical Neurosciences, University of CambridgeCambridge, UK; 2 Wolfson Brain Imaging Centre, Department of Clinical Neurosciences, University of CambridgeCambridge, UK; 3 Division of Anaesthesia, Department of Medicine, University of CambridgeCambridge, UK

**Keywords:** brain, human, microdialysis, pharmacokinetics, vigabatrin

## Abstract

**Aims:**

The aims were to determine blood–brain barrier penetration and brain extracellular pharmacokinetics for the anticonvulsant vigabatrin (VGB; γ-vinyl-γ-aminobutyric acid) in brain extracellular fluid and plasma from severe traumatic brain injury (TBI) patients, and to measure the response of γ-aminobutyric acid (GABA) concentration in brain extracellular fluid.

**Methods:**

Severe TBI patients (*n* = 10) received VGB (0.5 g enterally, every 12 h). Each patient had a cerebral microdialysis catheter; two patients had a second catheter in a different region of the brain. Plasma samples were collected 0.5 h before and 2, 4 and 11.5 h after the first VGB dose. Cerebral microdialysis commenced before the first VGB dose and continued through at least three doses of VGB. Controls were seven severe TBI patients with microdialysis, without VGB.

**Results:**

After the first VGB dose, the maximum concentration of VGB (*C*_max_) was 31.7 (26.9–42.6) μmol l^−1^ (median and interquartile range for eight patients) in plasma and 2.41 (2.03–5.94) μmol l^−1^ in brain microdialysates (nine patients, 11 catheters), without significant plasma–brain correlation. After three doses, median *C*_max_ in microdialysates increased to 5.22 (4.24–7.14) μmol l^−1^ (eight patients, 10 catheters). Microdialysate VGB concentrations were higher close to focal lesions than in distant sites. Microdialysate GABA concentrations increased modestly in some of the patients after VGB administration.

**Conclusions:**

Vigabatrin, given enterally to severe TBI patients, crosses the blood–brain barrier into the brain extracellular fluid, where it accumulates with multiple dosing. Pharmacokinetics suggest delayed uptake from the blood.

**What is Already Known About This Subject**

- Increasing the cerebral concentration of the inhibitory amino acid γ-aminobutyric acid (GABA) is putatively neuroprotective.
- The anticonvulsant vigabatrin (VGB) irreversibly inhibits GABA transaminase, thereby increasing GABA concentrations in brain.
- Vigabatrin blood and cerebrospinal fluid levels in epilepsy patients correlate poorly with biological effects of VGB, and it is unknown how efficiently VGB crosses the blood–brain barrier.

**What This Study Adds**

- This is the first study to address VGB penetration across the blood–brain barrier into the human brain extracellular fluid, in traumatic brain injury patients.
- Uptake of VGB into brain varies between patients, and within patients depending on injury location, suggesting blood–brain barrier differences.
- Uptake of VGB into brain extracellular fluid is slower than the VGB concentration rise in plasma after the first dose. Brain extracellular VGB concentration builds with multiple doses.

## Introduction

There is a dearth of neuroprotective drugs to alleviate the after-effects of traumatic brain injury (TBI). Many promising drugs in preclinical studies in animal models and in cell cultures have failed in clinical trials [1,2]. Possible reasons include differences in pharmacokinetics and pharmacodynamics between animal models and humans and poor blood–brain barrier (BBB) penetration of the drug. Another important factor is heterogeneity within the patient group, including variations in injury, genetic differences between patients and variable times of agent administration postinjury. Major questions are unanswered regarding whether drugs can reach the human brain at biologically relevant concentrations, at systemically acceptable doses.

Conventional pharmacokinetic (PK) studies usually focus on drug concentrations in plasma and cerebrospinal fluid (CSF). However, a more direct measure of the drug concentration to which brain cells are exposed involves determining the composition of the brain extracellular fluid (ECF), also termed interstitial fluid. The only feasible technology to date for sampling molecules from the human brain ECF is cerebral microdialysis [3]. This involves making an opening in the skull and inserting a fine catheter with a semi-permeable membrane (through which molecules can diffuse), perfusing the catheter with a ‘physiological’ saline solution and collecting the emerging solution, termed microdialysate, for analysis. Microdialysis works well for small water-soluble molecules. The most frequent implementation of clinical microdialysis is in neurocritical care monitoring of energy-related small molecules in the brain ECF, which has demonstrated abnormalities in brain metabolism after head injury that relate statistically to clinical outcome [4]. Clinical microdialysis catheters have nominal molecular weight cut-offs of 20 or 100 kDa. The technique is thus relevant to many drugs, although because it is invasive it can only be used in patients requiring neurocritical care, neurosurgery or brain biopsy. For example, microdialysis has been used successfully in clinical pharmacological studies of antibacterial agents in the brain [5,6].

We chose to investigate a putative neuroprotective drug; the anticonvulsant vigabatrin (VGB; γ-vinyl-γ-aminobutyric acid), a small, water-soluble molecule (129 Da) that binds irreversibly to γ-aminobutyric acid (GABA) transaminase (GABA-T) [7]. The inhibitory neurotransmitter GABA is degraded by GABA-T; blocking this enzyme increases the concentration of GABA, which is then thought to suppress abnormal electrical activity [8]. Vigabatrin given to epilepsy patients does increase GABA concentrations in CSF [9–11], and the CSF level of free GABA correlates with the CSF level of VGB [11]. However, there was no correlation between seizure frequency and CSF levels of GABA or VGB [11]. Also, no relationship was found between seizure control and serum VGB levels [12]. Therefore, it is difficult to use either serum or CSF to predict clinical response. A possible reason for the poor correlation between the clinical response to VGB and its concentration in serum or CSF is that these concentrations may differ from the VGB concentration in the brain ECF, which is the compartment to which the brain cells are directly exposed.

The aim of the present study, using VGB as a drug candidate, was to advance our understanding of drug transit across the BBB in severe TBI patients. The present study is part of our long-term objective to understand brain chemistry in TBI patients using cerebral microdialysis, which includes evaluating neuroprotective strategies. Vigabatrin is a good candidate molecule for microdialysis purposes because it is a small, hydrophilic molecule (low octanol/water partition coefficient), which is efficiently recovered by microdialysis (see Methods section). Being hydrophilic, the tendency of VGB to cross the intact BBB passively would be low. Furthermore, analysis of the microdialysates for the drug VGB itself, as well as GABA, a downstream marker, can be performed simultaneously by the same high-performance liquid chromatography (HPLC) method (see Methods section). Vigabatrin is predominantly not protein bound in plasma, not heavily metabolized by the liver, and is eliminated largely unchanged via the kidneys [13]; thus, it appears to be an ideal candidate drug for monitoring transit across the BBB into the brain ECF. We analysed the concentration (*vs*. time) of VGB in plasma and brain microdialysates of TBI patients (0.5 g VGB every 12 h). Also in brain microdialysates, we analysed concentrations of GABA, as a downstream marker. To our knowledge, this is the first study to address VGB penetration across the BBB into the human brain ECF, in the injured human brain.

## Methods

### Subjects

The study was approved by the Cambridge Local Research Ethics Committee and assent obtained from the next of kin. All subjects were patients in the Neurosciences Critical Care Unit (NCCU) in Addenbrooke's Hospital, Cambridge, UK. Patients were over 16 years of age with TBI, requiring ventilation and intracranial pressure monitoring. The major exclusion criterion was deranged clotting and/or low platelets, precluding the placement of a microdialysis catheter. None of the patients had any significant previous neurological conditions or family history of neurodegenerative disease. Ten TBI patients received VGB (Sabril [Aventis Pharma Ltd., West Malling, Kent, UK]; 0.5 g every 12 h, enterally); seven TBI patients not receiving VGB acted as controls.

### Bioanalytical sampling and assays

#### Blood sampling

Blood samples were taken from the VGB patients 0.5 h before the first dose was given, and then again at 2, 4 and 11.5 h after the first dose. No later blood samples were taken. Proteins were removed from the plasma samples by ultrafiltration (Sigma M0911, 10 kDa molecular weight cut-off; Sigma-Aldrich, Poole, Dorset, UK) and the clear, colourless ultrafiltrates were diluted, using a ratio of 5 μl of ultrafiltrate to 15 μl water, then analysed for VGB by HPLC as described below.

#### Microdialysis

All patients were monitored using microdialysis, which was started as soon as possible following admission to the NCCU. Microdialysis catheters (CMA71 or CMA70, 100 or 20 kDa molecular weight cut-off, respectively, 10 mm membrane; M Dialysis AB, Solna, Sweden) were inserted into the cerebral frontal parenchyma of patients, together with an intracranial pressure transducer (Codman, Raynham, MA, USA) using a triple-lumen cranial access device (Technicam, Newton Abbot, Devon, UK). In two VGB patients and one control patient, a second microdialysis catheter was inserted via a craniotomy site. The catheters were perfused with central nervous system (CNS) perfusion fluid (NaCl, 147 mmol l^−1^; KCl, 2.7 mmol l^−1^; CaCl_2_, 1.2 mmol l^−1^; and MgCl_2_, 0.85 mmol l^−1^ in water; M Dialysis AB) at 0.3 μl min^−1^ using a CMA106 pump (M Dialysis AB). Collection vials were changed hourly and analysed at the bedside for glucose, lactate, pyruvate and glutamate or glycerol, using a CMA600 microdialysis analyser (M Dialysis AB) using automated enzymatic colorimetric assays, as part of routine multimodality monitoring. Microdialysates were then stored at −80°C for subsequent analysis by HPLC to determine VGB and GABA. Not all vials had sufficient microdialysate volume remaining for HPLC analysis and, in some cases (e.g. when long sequences were being evaluated), microdialysates from two vials were pooled for HPLC analysis.

#### *In vitro* microdialysis recovery experiment

Recovery of VGB was determined *in vitro* by immersing the tip of the microdialysis catheter (CMA 71, 100 kDa cut-off) into 20 ml of a solution of VGB (25 μmol l^−1^) in CNS perfusion fluid, at 37°C. The catheter was perfused with CNS perfusion fluid at 0.3 μl min^−1^ using a CMA106 pump. Microdialysate collection vials were changed every 30 min for a period of 5 h and stored at −80°C for subsequent analysis by HPLC using the same method as for brain microdialysates (see below). The percentage recovery (extraction efficiency) of VGB was calculated as the concentration of VGB in the microdialysate divided by the concentration of VGB in the external solution × 100%, giving a mean (±SD) of 103.6% (±4.3%). Vigabatrin recovery was thus considered 100%. Brain microdialysate VGB concentrations were therefore reported as measured and not adjusted; likewise for GABA and other amino acids.

#### Determination of VGB and GABA concentration by HPLC

Microdialysate samples were diluted using the ratio of 5 μl of microdialysate to 10 μl of water prior to HPLC analysis on an Agilent 1100 series HPLC (Agilent Technologies, Waldbronn, Germany), using methodology published previously [14,15]. The HPLC system comprised a binary pump, refrigerated autosampler (at 10°C) and fluorescence detector (at 340 nm excitation, 450 nm emission), with a ChemStation data system. The column (maintained at 40°C) was a Phenomenex Luna C18(2) (100 mm × 2 mm, particle size 3 μm, pore size 100 Å; Phenomenex, Torrance, CA, USA), with a Phenomenex SecurityGuard Luna C18(2) guard cartridge. The mobile phase (0.45 ml min^−1^) was continuously vacuum degassed. Using the autosampler, each prediluted microdialysate sample (1 μl) was automatically mixed in-needle with 5 μl of borate buffer (Agilent; 0.4 mol l^−1^, pH 10.2), 1 μl of OPA reagent (Agilent; 10 mg ml^−1^ *ortho*-phthalaldehyde in 0.4 mol l^−1^ borate buffer plus mercaptopropionic acid) and 1 μl of internal standard (25 μmol l^−1^ norvaline) and injected onto the HPLC column. Separation was achieved using gradient elution. Mobile phase A was composed of 20 mmol l^−1^ sodium acetate in water, 107 mmol l^−1^ EDTA, plus 180 μl of triethylamine and 3 ml of tetrahydrofuran per litre, pH 7.2. Mobile phase B consisted of a 1:2:2 (v/v/v) mixture of 20 mmol l^−1^ sodium acetate in water (pH 7.2), methanol and acetonitrile. Initial conditions (at time of injection) were 100% A and 0% B, changing linearly over a period of 17 min to 40% A and 60% B, which was maintained isocratically until 25 min after injection, then returned to initial conditions by 30 min after injection, with a 5 min postrun equilibration period before the next injection. Quantification was by peak areas relative to the norvaline internal standard, with reference to an external standard mixture of amino acids (Agilent, part no. 5061-3331) plus norvaline, GABA and VGB (Sigma-Aldrich, Poole, Dorset, UK). The drug VGB is a racemic (50:50) mixture of two enantiomers; *S*(+) is the pharmacologically active form and *R*(−) inactive. In common with the majority of other studies, our methodology measured total VGB and did not distinguish between enantiomers. Other amino acids of interest, analysed alongside VGB within the same HPLC run, were GABA, glutamic acid, serine, threonine, tyrosine and leucine.

The precision of the HPLC method (relative standard deviation, i.e. coefficient of variation) was 0.6–5.1% for 16 standard amino acids and the limit of detection 16–57 fmol injected onto the HPLC column [15]. In brain microdialysates, the working lower limit for VGB was ∼0.4 pmol injected (corresponding to 1.2 μmol l^−1^ in the microdialysate) due to an unknown substance with a similar retention time to VGB. This unknown was present in microdialysates from control patients who did not receive VGB, as well as in microdialysates collected before the first dose in patients who subsequently received VGB.

Brain microdialysates from each patient were individually analysed for a pre-dose monitoring period typically lasting 4–8 h to establish a baseline. Microdialysates for a period of time covering at least three doses of VGB were then analysed individually. The time quoted for each microdialysis sample corresponds to the time after the first dose when each collection vial was removed from the microdialysis catheter.

### Pharmacokinetic assessments for VGB

Plasma samples were available for the first dose only. The maximum concentration in plasma (*C*_max,pl_) was determined directly from the measured value at either 2 or 4 h. The time of maximum concentration in plasma (*T*_max,pl_) could not be determined with precision from the four plasma measurements. The area under the vigabatrin concentration–time curve in plasma from zero to 11.5 h (AUC_0–*t*,pl_) for the first dose was determined with the linear trapezoids rule using the four plasma measurements at 0, 2, 4 and 11.5 h.

Microdialysis data were available from several hours before the first dose to at least the end of the third dose (i.e. 36 h after the first dose) in most VGB patients. The maximum concentration in brain and time of maximum concentration in brain after the first (*C*_max,br1_, *T*_max,br1_) and third doses (*C*_max,br3_, *T*_max,br3_) were determined directly from the VGB microdialysis data. The area under the VGB concentration–time curve in brain for the first (AUC_0–*t*,br1_) and third doses (AUC_0–*t*,br3_) were determined with the linear trapezoids rule using the VGB measurements between 0 and 12 h after the time of the dose, or as close to these timings as the measurements allowed. The average VGB concentrations for the first and third doses (*C*_av,br1_, *C*_av,br3_) were calculated by dividing the corresponding AUC value by the total time used for AUC measurement. The average pre-dose concentration (*C*_av,pre_) of the unknown substance that co-elutes with VGB, present in most microdialysates before VGB dosing, was calculated from the pre-dose AUC value. The accumulation ratio (*R*_ac_) was calculated as *C*_av,br3_/*C*_av,br1_.

The seven control TBI patients without VGB did not receive a placebo. Microdialysates from each control patient (one patient with two catheters) were analysed for VGB, GABA and other amino acids by HPLC over a representative 48 h period beginning 2 days after commencement of microdialysis, corresponding to the average day of first dose for the VGB patients. An unknown substance co-eluting with VGB, present in microdialysates from seven of the eight catheters, was quantified as for VGB for the first and third 12 h periods of monitoring. The area under the VGB concentration–time curve (AUC_0–*t*,control1_ and AUC_0–*t*,control3_) for this unknown were calculated as described for the VGB patients above.

Data analysis and PK calculations were carried out using Microsoft Excel. Statistical analysis was carried out using StatView (SAS Institute Inc., Cary, NC, USA; version 5). Student's paired *t* tests were used to compare mean microdialysate concentrations between groups. Correlations between parameters were determined using Spearman correlation coefficients or simple linear regression analysis. Statistical significance was considered at *P* < 0.05.

## Results

Ten patients who received VGB and seven control (non-VGB) patients were recruited into the study. Patient demography is presented in Supporting Information Table S1. The data for one of the VGB patients were excluded from the results due to uncertain timing of doses (Patient 5). For the VGB patients, the mean (±SD) time of first VGB dose after injury was 3.6 ± 1.0 days. For the control patients, the time of first HPLC analysis after injury was 3.6 ± 1.7 days.

### First-dose vigabatrin pharmacokinetics for uptake from blood into brain

For eight of the 10 VGB patients, blood plasma samples for VGB analysis were collected 0.5 h before the first dose and at 2, 4 and 11.5 h after the first dose. Of the two remaining VGB patients, one had no available blood samples (Patient 9) and the other (Patient 5) was excluded as described above.

**Figure 1:**
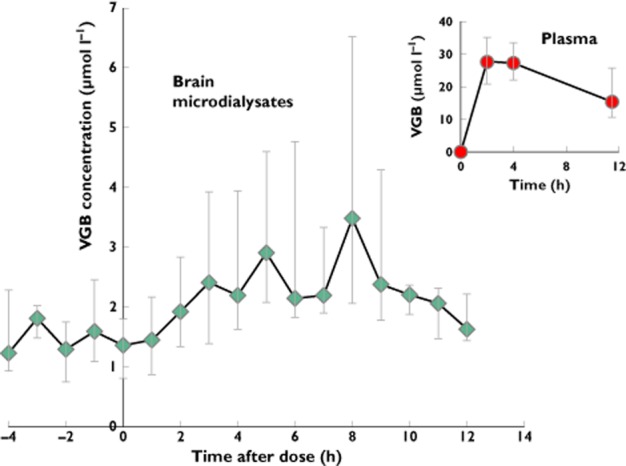
Median [and interquartile range (IQR)] vigabatrin (VGB) concentrations (μmol l^−1^) plotted *vs.* time (in hours) in brain microdialysates (main graph) and plasma (inset) in relation to the first dose. Microdialysate VGB concentration data for nine VGB patients were pooled into hourly periods. Plasma VGB concentrations were for eight patients at 2, 4 and 11.5 h after the first dose. The number of microdialysate samples (*n*) for each hourly time point is as follows: −4 h (5); −3 h (4); −2 h (5); −1 h (9); 0 h (8); 1 h (9); 2 h (8); 3 h (10); 4 h (9); 5 h (8); 6 h (8); 7 h (8); 8 h (8); 9 h (7); 10 h (6); 11 h (6); 12 h (5)

The time course of VGB concentration, expressed as medians (interquartile range, IQR), in plasma and brain microdialysates is shown in Figure 1. Following the first dose, VGB rose rapidly in plasma, reaching 27.5 (20.8–35.1) μmol l^−1^ by 2 h and 27.2 (22.1–33.4) μmol l^−1^ by 4 h and then falling to 15.3 (10.6–25.8) μmol l^−1^ by 11.5 h. Key plasma PK values are shown in Table 1 as medians (IQR), with individual results in Supporting Information Table S2. The data are subject to the limitations of sampling times and, consequently, the first-order rate constant for elimination of VGB from plasma could not be determined.

**Table 1:** Pharmacokinetic results [median (interquartile range)] for vigabatrin in blood plasma and brain microdialysates

	*C*_max_ (μmol l^−1^)	*T*_max_ (h)	AUC_0–_*_t_* (h μmol l^−1^)
**Plasma**			
First VGB dose (eight patients*)	31.7 (26.9–42.6)	3.0 (2.0–4.0)	249.1 (205.4–317.3)
**Brain microdialysates**			
** First VGB dose (nine patients, total 11 catheters)**	2.41 (2.03–5.94)	5.1 (4.3–7.4)	23.2 (17.0–41.0)
** Third VGB dose (eight patients**†**, total 10 catheters)**	5.22 (4.24–7.14)	4.7 (4.0–5.6)	43.8 (36.4–58.8)

Abbreviations are as follows: AUC, area under curve; *C*_max_, maximum concentration; *T*_max_, time of maximum concentration; VGB, vigabatrin. The VGB patients received 0.5 g VGB every 12 h.

* One of the patients did not have blood samples taken (Patient 9).

† Brain microdialysates of one of the VGB patients (Patient 3) were not monitored up to the third VGB dose.

See Supporting Information Tables S2 and S3 for VGB data for individual patients. The *C*_max_ values for brain microdialysates include a contribution from an unknown species with the same high-performance liquid chromatography retention time as VGB, which was equivalent to a median (interquartile range) of 1.19 (0.61–1.93) μmol l^−1^ VGB (*C*_av,pre_), quantified from the pre-first dose period for the VGB patients. Control patients did not receive VGB and had no blood samples taken. The unknown species was present in microdialysates from control patients (seven patients, eight catheters) and had AUC_0–_*_t_* values of 9.43 (7.0–18.2) and 14.9 (11.0–17.9) h μmol l^−1^ for the first and third 12 h monitoring periods, respectively.

**Figure 2:**
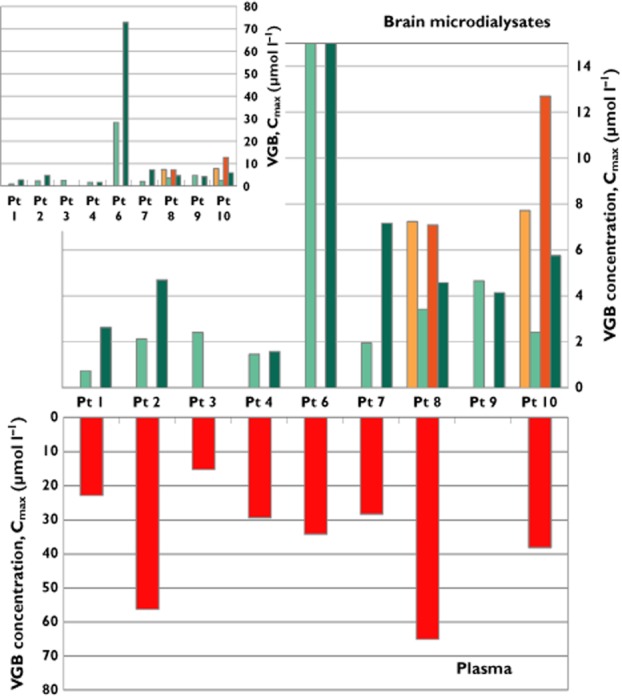
Maximum VGB concentrations (*C*_max_) measured in brain microdialysates (first and third doses; upper main plot), and in plasma samples (first dose; lower plot), expressed as μmol l^−1^. Inset shows microdialysate results for Patient 6 in full scale. Each patient had a microdialysis catheter inserted via a cranial access bolt (B) device; Patients 8 and 10 also had another (A) catheter inserted via a craniotomy site. Brain microdialysate results after the first and third doses are shown in light and dark green for the B catheters, and light and dark orange for the A catheters, respectively. Plasma samples were collected after the first dose only (results in red). No plasma samples were available for Patient 9. Microdialysates for Patient 3 were not monitored past the second dose. 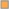, first dose (A); 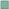, first dose (B); 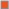, third dose (A); 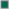, third dose (B)

**Figure 3:**
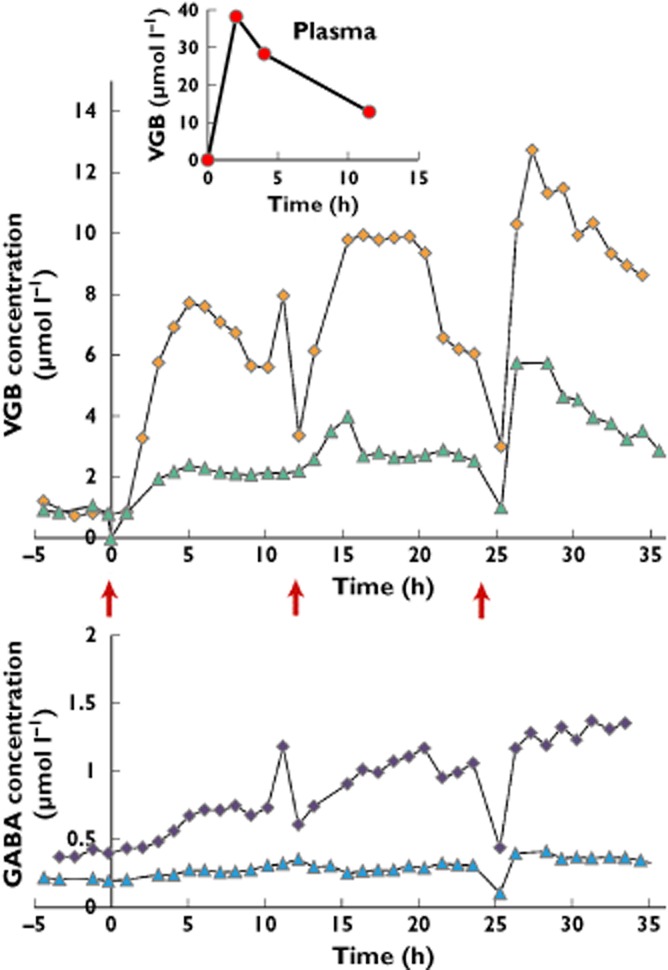
Illustrative case example: Patient 10. Vigabatrin concentrations (μmol l^−1^) plotted *vs.* time (in hours), for brain microdialysates and plasma, and the corresponding γ-aminobutyric acid (GABA) concentrations in brain microdialysates, for the first, second and third VGB doses given at the times indicated by red arrows. Upper panel shows VGB concentrations in microdialysates from catheters A (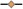) and B (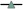). Inset: VGB concentrations in plasma (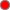). Lower panel: GABA concentrations in microdialysates from catheters A (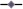) and B (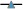)

First-dose VGB concentrations in brain microdialysates were highly variable between patients and much lower than plasma VGB concentrations (Table 1 and Figure 2). There was no significant correlation between microdialysate and plasma VGB concentrations (Spearman *r* = 0.53, *P* = 0.11; Supporting Information Figure S1). Pharmacokinetic values for VGB in brain microdialysates are shown in Table 1 as medians (IQR), with individual patient values in Supporting Information Table S2. The maximum VGB concentration in brain microdialysates after the first dose (*C*_max,br1_), shown in Figure 2 for each patient, had a median (IQR) of 2.41 (2.03–5.94) μmol l^−1^, with a full range of 0.74–28.3 μmol l^−1^. The values of *C*_max_ include a background component from a co-eluting unknown substance in the HPLC chromatograms, equivalent to a median (IQR) of 1.19 (0.61–1.93) μmol l^−1^ VGB (*C*_av,pre_), quantified from the pre-first dose period for the VGB patients. Presented in Supporting Information Table S3, the individual patients' *C*_av,pre_ levels were always lower than their corresponding post-dose VGB concentrations in microdialysates. Patients 2 and 4 showed negligible increase in microdialysate VGB concentration above baseline after the first dose. Patient 6, whose microdialysis catheter was in abnormal brain, had a more rapid and significant VGB uptake into the brain than the others, reaching a *C*_max_ of 28.3 μmol l^−1^ at 3.2 h after the first dose. In the patients with two catheters each, the VGB concentration was greater at the site closer to focal injury (catheter A) compared with the more distant site (catheter B). Patient 10 had *C*_max,br1_ values of 7.7 and 2.41 μmol l^−1^ in catheters A and B, respectively, while plasma *C*_max,pl_ was 38.1 μmol l^−1^ (Figure 3). Patient 8 had *C*_max,br1_ values of 7.23 and 3.41 μmol l^−1^ in catheters A and B, respectively; plasma *C*_max,pl_ was 65.1 μmol l^−1^.

The area under the VGB concentration–time curve in microdialysates between 0 h and the time of the last measurement closest to 12 h for the first dose (AUC_0–*t*,br1_) was determined for each VGB patient, giving a median (IQR) of 23.2 (17.0–41.0) h μmol l^−1^, with full range 2.2–181.0 h μmol l^−1^. The AUC includes a background unknown component that co-elutes with VGB (see above). This unknown was also present in microdialysates from the control patients who received no VGB and had a median (IQR) AUC of 9.43 (7.0–18.2) h μmol l^−1^ for the first 12 h monitoring period (AUC_0–*t*,control1_). Individual patient values for *C*_max_ and AUC are shown in Supporting Information Table S2.

The time course of VGB uptake into the brain varied between patients. Figure 1 shows the median (IQR) microdialysate VGB concentrations for nine patients pooled into hourly periods after the first dose. The overall pattern showed that VGB concentration rose to a maximum at ∼8 h, then decreased to a minimum by 12 h. Individual values for the time of maximum concentration of VGB in brain after the first dose (*T*_max,br1_) are shown in Supporting Information Table S2, giving a median (IQR) of 5.1 (4.3–7.4) h, with a full range of 3.2–9.8 h. Due to clinical dosing constraints of repeat doses of VGB being given at 12 h intervals, we were unable to obtain accurate VGB elimination pharmacokinetics from brain.

### Multiple dose accumulation of vigabatrin in brain

Following the first dose, VGB doses (0.5 g) were repeated at 12 h intervals, with microdialysis monitoring but without further plasma sampling. For eight patients receiving VGB, brain microdialysates were monitored to at least the end of the third dose (i.e. 36 h after the first dose). The maximum VGB concentration in brain microdialysates after the third dose (*C*_max,br3_) had a median (IQR) of 5.22 (4.24–7.14) μmol l^−1^. As for the two patients who had negligible brain uptake of VGB after the first dose, only Patient 4 still had minimal increase in microdialysate VGB concentration above baseline after the third dose, while Patient 2 showed a typical increase above baseline.

The accumulation ratio, *R*_ac_, calculated here as the average VGB concentration after the third dose (*C*_av,br3_) divided by the average VGB concentration after the first dose (*C*_av,br1_), had a median (IQR) of 1.8 (1.2–2.6), with full range 1.0–4.6, in eight patients (10 catheters; Supporting Information Table S3). As well as interpatient variability, there were apparent differences in *R*_ac_ at different sites within the brain, suggesting the influence of injury on BBB permeability, with B sites greater than A, although only two patients had A and B paired catheters. In Patient 10, *R*_ac_ for catheters A and B were 1.64 and 2.05, respectively, while in Patient 8 the corresponding values were 1.1 and 1.6.

### γ-Aminobutyric acid and amino acid concentrations in brain microdialysates

**Figure 4:**
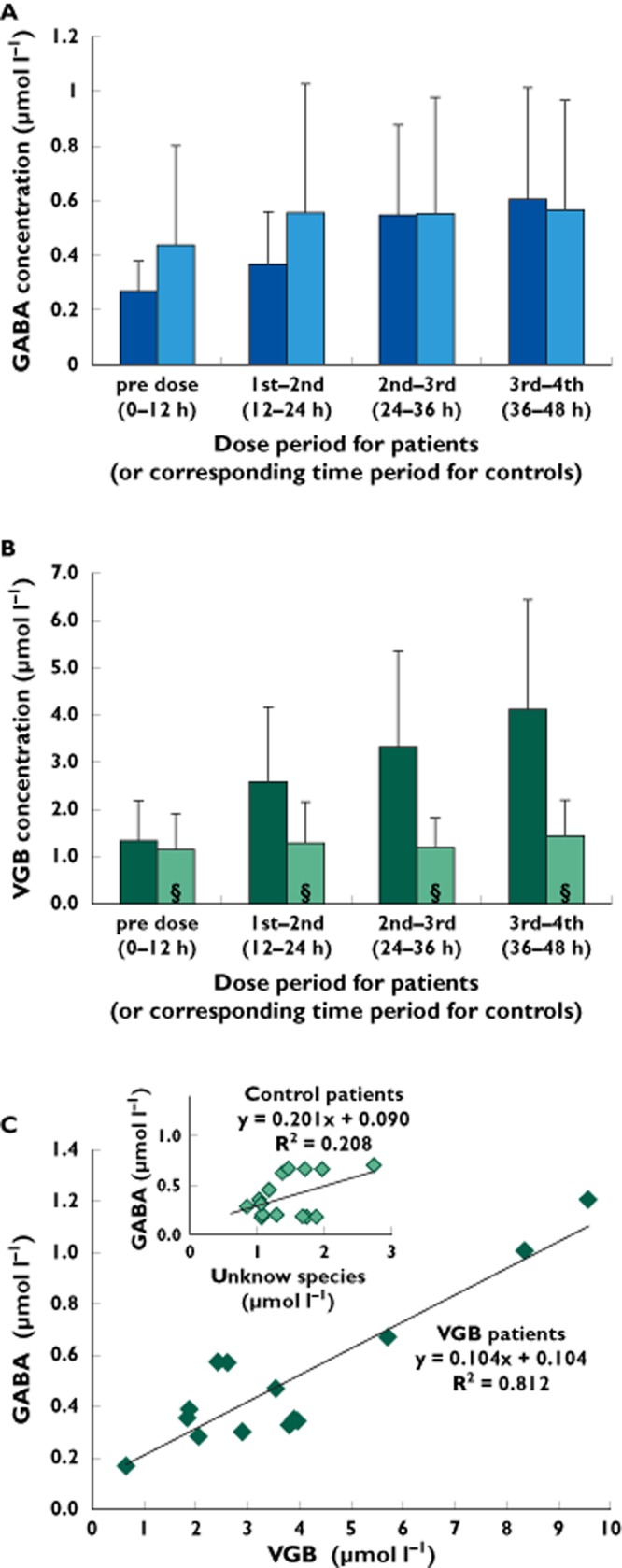
(A) Brain microdialysate GABA concentrations (μmol l^−1^; means ± SD) in VGB patients (Patients 1, 3, 9, 10A and 10B) and control patients (Patients 11, 12, 13, 14, 15B, 16 and 17). Data were combined into four time periods, comprising a pre-dose period plus three inter-dose periods (first–second dose, second–third dose and third–fourth dose) in the VGB patients, or to four corresponding time periods in the control patients (without VGB). The difference between GABA concentrations in the pre-dose and the first–second inter-dose period approached but did not achieve statistical significance (*P* = 0.06). Other comparisons were not statistically significant (*P* > 0.1). (B) Brain microdialysate VGB concentrations (μmol l^−1^; means ± SD) in VGB patients (Patients 1, 2, 3, 4, 7, 8A, 8B, 9, 10A and 10B) and in control patients (Patients 11, 12, 13, 14, 15A, 15B, 16 and 17) the concentrations of the unknown species that eluted with the same high-performance liquid chromatography (HPLC) retention time as VGB, expressed as μmol l^−1^ equivalent to VGB (above axis, § denotes unknown rather than VGB concentration). (C) Main graph shows a bivariate scattergram of brain microdialysate concentrations of GABA (μmol l^−1^) plotted *vs.* brain microdialysate concentrations of VGB (μmol l^−1^) for the VGB patients in (A). Pre-dose data were omitted. Each data point represents one catheter for one time period (defined in A). There was a statistically significant linear relationship between GABA and VGB concentrations (*P* < 0.0001). Inset graph shows a bivariate scattergram of brain microdialysate concentrations of GABA (μmol l^−1^) plotted *vs.* brain microdialysate concentrations of the unknown species (μmol l^−1^) that eluted with the same HPLC retention time as VGB for the control patients in (A). Data for the first period (equivalent to pre-dose) were omitted. Data points were omitted if GABA and/or the unknown species were undetectable. Each data point represents one catheter for one time period (defined in A). There was no statistically significant linear relationship between GABA concentrations and those of the unknown species (*P* = 0.09). (a) 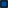, VGB patients; 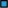, control patients (b) 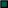, VGB patients; 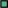, control patients

**Figure 5:**
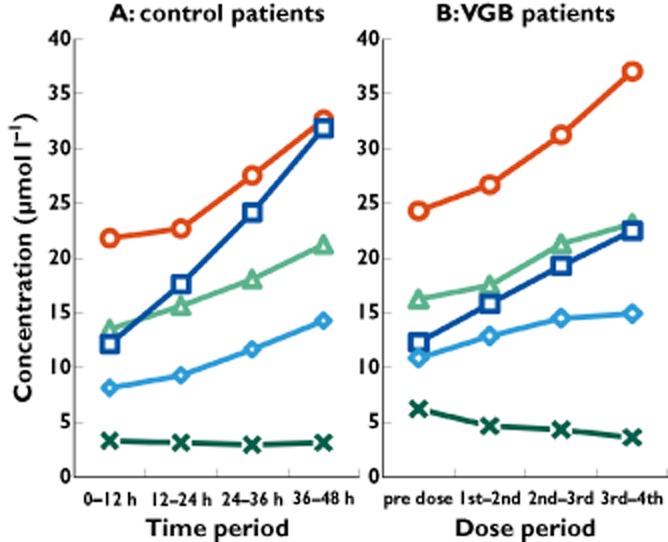
Amino acid concentrations (μmol l^−1^) in brain microdialysates from control patients and VGB patients, plotted *vs.* time periods for VGB doses, or the corresponding time periods for the control patients without VGB. 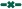, glutamic acid; 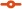, leucine; 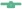, serine; 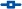, threonine; 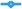, tyrosine

We determined microdialysate concentrations of GABA, glutamic acid, serine, threonine, tyrosine and leucine, within the same HPLC run as VGB. The average microdialysate GABA and amino acid concentrations were calculated for the following four time periods: pre-dose; between first and second doses; between second and third doses; and between third and fourth doses. The pre-dose monitoring period median (IQR) was 4.2 (3.0–5.3) h, and the periods between doses were ∼12 h each. For the control patients (no VGB), the 48 h of microdialysis monitoring was subdivided into four 12 h periods, and the average GABA and amino acid concentrations calculated for each period were compared with values for the patients receiving VGB over the corresponding periods of time. Results are shown in Figures 4 and 5.

Consistent with earlier microdialysis work on non-VGB patients [16], the baseline concentration of GABA in brain microdialysates was very low or, in some cases, undetectable, both pre-dose in the patients who subsequently received VGB and in the non-VGB control patients. The first VGB dose effect on microdialysate GABA concentration was slight. Microdialysate GABA concentrations were measurable up to the end of the third dose in VGB Patients 1, 9 and 10 (catheters A and B) and up to the end of the second dose in Patient 3. Mean GABA concentration (±SD) increased steadily from 0.27 ± 0.11 μmol l^−1^ before the first dose to 0.61 ± 0.30 μmol l^−1^ for the period between the third and fourth doses (Figure 4A), although this increase and the increases for each inter-dose period did not reach statistical significance (Student's paired *t* test; *P* > 0.05). The largest increase in GABA over the course of three doses was seen in Patient 10, catheter A, from 0.43 μmol l^−1^ before the first dose to 1.21 μmol l^−1^ after the third dose (Figure 3). The concentration of GABA was measurable in microdialysates from six control patients. There was a greater increase (relative to pre-dose baseline) in mean microdialysate GABA level over time in the VGB patients than in the control (non-VGB) patients (Figure 4A).

The mean VGB concentration for all VGB-treated patients for each of the above time periods was also calculated so that an overall relationship between microdialysate VGB and GABA levels could be determined. The mean VGB concentration (±SD) for all VGB-treated patients (except Patient 6) increased from 1.34 ± 0.84 μmol l^−1^ before the first dose to 2.57 ± 1.59 μmol l^−1^ after the first dose and 4.12 ± 2.32 μmol l^−1^ after the third dose (Figure 4B). The increase from the pre-dose period to the first–second inter-dose period was statistically significant (Student's paired *t* test; *P* = 0.03), as was the increase from the first–second inter-dose period to the third–fourth inter-dose period (*P* = 0.008). There was a significant positive linear relationship (*P* < 0.0001) between GABA and VGB concentrations in microdialysates from VGB patients (Figure 4C, main graph). Vigabatrin concentrations were higher than those of the unknown species with the same HPLC retention time as VGB in control patients (Figure 4B). The mean concentration (±SD) of the unknown species in all control patients for the first 12 h period was 1.15 ± 0.76 μmol l^−1^, which increased slightly to 1.28 ± 0.87 μmol l^−1^ for the second 12 h period and 1.43 ± 0.77 μmol l^−1^ for the final monitoring period. Neither of these increases was statistically significant compared with the mean concentration for the first 12 h period. There was not a significant linear relationship between GABA and the unknown species in the microdialysates of control patients (*P* = 0.09; Figure 4C, inset graph).

The concentrations of serine, threonine, tyrosine and leucine showed steady increases with time in both VGB patients and control patients (Figure 5). For the control patients, the mean concentration of these four amino acids increased significantly between the first 12 h period and the final period of monitoring. For the VGB patients, the increase from the pre-dose period reached significance by the third–fourth inter-dose period for threonine, tyrosine and leucine, but not serine. Glutamic acid did not change in the control patients with time, and showed a modest decrease in the VGB patients but without statistical significance (Figure 5).

## Discussion

This study is the first to address VGB penetration across the BBB into the human brain ECF in TBI patients, using cerebral microdialysis, after enteral administration of 0.5 g doses. A preliminary report illustrating VGB concentrations *vs*. time in brain microdialysates from three patients from this cohort did not include PK calculations [14]. The microdialysis technique enables the brain ECF to be sampled continuously, allowing determination of drug PK in the brain. Other researchers' PK studies have measured VGB and GABA in the CSF of patients with epilepsy [9,11,17–19]. However, the CSF is a different compartment of the brain, separated from the blood by a different barrier, so concentrations in CSF and brain ECF are not necessarily the same [20,21]. The brain ECF concentration of a drug is likely to determine its therapeutic potential, because this is the compartment to which neurons and astrocytes are directly exposed.

The degree of uptake of VGB into the brain ECF appeared highly variable and did not correlate with VGB concentration in blood. This highlights a central issue in CNS drug discovery, i.e. the transport of drugs across the BBB. An important issue arising from this study is to what degree the variability in VGB brain concentration is due to differences in brain injury between patients or to an inherent variability in the general population. Evidence from the two patients who had two catheters each (catheter A closer to and catheter B further from a focal lesion) showed that VGB concentration was greater at sites closer to damaged brain, suggesting that BBB permeability plays a role.

### Plasma pharmacokinetics of vigabatrin in TBI patients compared with healthy subjects

The TBI patients' first-dose VGB results in plasma, with a median (IQR) *C*_max,pl_ of 31.7 μmol l^−1^ (IQR, 26.9–42.6 μmol l^−1^, *n* = 8; Table 1), were lower than those reported by Hoke *et al*. in healthy volunteers (*n* = 24), whose mean (± SD) *C*_max_ was 133.9 ± 20.9 μmol l^−1^ (originally reported as 17.3 ± 2.7 μg ml^−1^) after being given the first oral 0.5 g dose following overnight fasting [22]. However, our *C*_max,pl_ values in TBI patients might not accurately reflect the true peak VGB concentration in plasma, due to the limited blood-sampling schedule. The area under the plasma VGB concentration–time curve for our TBI patients during 0–11.5 h had a median (IQR) of 249.1 (205.4–317.3) h μmol l^−1^. A higher mean (±SD) AUC_0–12h_ was reported by Hoke *et al*. in plasma of healthy volunteers as 439.8 ± 49.6 h μmol l^−1^ (56.8 ± 6.4 h μg ml^−1^) after the first 0.5 g VGB dose [22]. Although we could not precisely determine the time of maximum VGB concentration in plasma (*T*_max,pl_), because our plasma sampling times were limited to pre-first dose and 2, 4 and 11.5 h post-first dose, it is noteworthy that four of the eight TBI patients had higher plasma VGB concentrations at 4 h than at 2 h, suggesting that the *T*_max,pl_ in some patients is later than the average 1–2 h reported in healthy volunteers [22–25]. Potentially, the slower absorption rates in the enterally fed TBI patients may contribute to their lower *C*_max,pl_.

### Factors influencing brain levels of vigabatrin

The concentration of VGB in brain microdialysates after the first dose varied widely between patients (Figures 1 and 2) and showed no significant correlation with plasma concentration (Supporting Information Figure S1). Brain permeability can be described by the brain-to-plasma maximum concentration ratio *C*_max,br_/*C*_max,pl_, for each patient (Supporting Information Table S2). Notably, this ratio is subject to uncertainty in *C*_max,pl_ owing to the limited blood-sampling times (see above). Patient 6, whose catheter was in abnormal brain, had a high *C*_max,br_/*C*_max,pl_ ratio of 0.83. In contrast, the values of *C*_max,br_/*C*_max,pl_ for the other patients were much lower (full range 0.03–0.20), with A catheters (closer to a focal lesion) having higher values than B catheters (distant from a focal lesion; Supporting Information Table S2). Brain extracellular VGB concentration is evidently influenced by other factors besides plasma concentration, which could include the following: (i) the extent of BBB permeability or damage, changing the rate of passive diffusion from blood to brain; (ii) the rate of VGB uptake into brain cells; and (iii) the rate of removal of VGB from the brain ECF by efflux transporters. Whether differences in these factors are a consequence of the severity of injury or due to inherent differences between patients and different regions of the brain is as yet unknown. We have limited information regarding the transport of VGB across the BBB and cell membranes. Vigabatrin is not a substrate for the multidrug transporter P-glycoprotein, which is implicated in the efflux of other antiepileptic drugs from the brain [26,27]. While the literature suggests that VGB has interactions with other transporters, the roles of these transporters in the context of the brain are poorly understood and are outside the scope of the present article.

The present study enabled site-specific comparison of VGB behaviour within the brain. Patients 8 and 10 had two catheters inserted respectively near to (catheter A) and further from a focal lesion (catheter B). Although *C*_max,br1_ differed markedly in the two sites, the time of peak concentration (*T*_max,br1_) was 5.1 h in both catheters A and B for Patient 10 (Figure 3), while in Patient 8 the *T*_max,br1_ was 5.7 h in catheter A and 4.7 h in catheter B (Supporting Information Table S2). These similarities in timing between sites A and B might imply some similarities in transport mechanism across the BBB. The differences in concentration (A > B) could be due to several factors, as discussed above. More patients with A and B paired catheters would be needed in order to elucidate site-specific differences further.

Brain uptake of several other antiepileptic drugs in epilepsy, studied by Rambeck *et al*., has shown not only a large variability in brain uptake of a particular drug between patients, but also intrapatient variability when two or more catheters are placed in locations within the epileptogenic zone [21].

The integrity of the BBB in the region of the catheter will depend on the type of injury (diffuse or local) and presumably also on co-morbidities and co-medications. Determination of BBB integrity at the site of each catheter was not carried out in the present study. However, a possibly relevant observation may be the increase (with time) in microdialysate levels of several amino acids (leucine, serine, threonine and tyrosine) seen in both VGB patients and control patients (Figure 5). None of the four amino acids is a neurotransmitter *per se*, and two of them (leucine and threonine) are essential amino acids that humans cannot synthesize; therefore, an increase in microdialysate levels of amino acids with time may reflect changing BBB permeability. The pattern of individual patients' first-dose data for these microdialysate amino acid concentrations (Supporting Information Figure S2) is similar but not identical to that of first-dose VGB uptake (Figure 2), adding to evidence that BBB permeability may be an important factor in VGB uptake.

Although we could not determine with precision the time of maximum VGB concentration in plasma (*T*_max,pl_) owing to the limited number of post-dose blood-sampling time points (2, 4 and 11.5 h), all eight patients with available data showed their highest VGB plasma concentrations at 2 or 4 h. In brain microdialysates, VGB median (IQR) *T*_max,br1_ was 5.1 (4.3–7.4) h. These data suggest that brain extracellular VGB peaks at a similar or later time to plasma. A delay between peak concentration in plasma and peak concentration in brain was described by Tong *et al*. in a microdialysis study of vigabatrin pharmacokinetics in rat brain and plasma [28], and was probably due to distributional processes across the BBB. In a pharmacokinetic study of morphine in rats, Bouw *et al*. were able to conclude that 85% of the delay between the plasma concentration of morphine and the antinociceptive effect of the drug could be explained by transport across the BBB [29].

### Multiple-dose accumulation of vigabatrin in brain

Eight of the TBI patients were monitored by microdialysis sampling up to the end of the third dose, i.e. 36 h after the first dose. The brain microdialysate values for *C*_max_ and AUC for the third VGB dose were about twice those for the first dose, showing marked accumulation of the drug in the brain. However, as discussed above, it is not known how much of this increase might have been due to increased BBB permeability and how much might have stemmed from a possible build-up in plasma VGB concentration with repeated dosing (note that we measured plasma VGB only after the first dose). Other researchers have reported plasma VGB levels reaching a plateau after three VGB doses in healthy volunteers [22]. In the TBI patients in the present study, the accumulation ratio, *R*_ac_, in brain ECF, calculated from the average VGB concentrations for the third and first doses, had a median of 1.8 and varied between patients (full range 1.0–4.6). The A catheters of Patients 8 and 10 had lower *R*_ac_ than the B catheters, suggesting that the rate of efflux may be dependent on local BBB disruption. Moreover, BBB permeability might change with time, and temporal changes might differentially affect sites A and B. More patients with two catheters would be needed for definitive evaluation.

### Microdialysis compared with cerebrospinal fluid sampling

The continuous nature of microdialysis is an advantage over CSF sampling for measuring changes with time. Cerebrospinal fluid drainage was not performed in the TBI patients in our study, because it was not clinically appropriate in these individuals. Therefore, we compare our microdialysates with CSF data from the literature, as follows. Ben-Menachem *et al*. studied 11 epilepsy patients, each given a single 50 mg kg^−1^ oral VGB dose [30]. Mean maximum VGB concentration in plasma was ∼500 μmol l^−1^ after 1 h in these epilepsy patients [30]. The VGB concentration in the CSF of the epilepsy patients, measured at 6, 24, 72, 120 and 168 h, was highest at 6 h, and had fallen almost to zero by 24 h [30]. The VGB concentration in the CSF of epilepsy patients at 6 h (mean ± SD, 11.5 ± 2.5 μmol l^−1^) [30] was higher than the median *C*_max,br_ measured in the present study in the brain microdialysates of the TBI patients after the first dose (2.41 μmol l^−1^). Possible explanations may include differences in pathology between TBI and epilepsy and/or the larger dose in the epilepsy patients (50 mg kg^−1^ dose equates to 3.35 g, based on mean bodyweight of the epilepsy subjects) [30] compared with the present TBI study (0.5 g). Inherent differences may exist between the ECF and CSF compartments, e.g. more effective efflux transport from the brain ECF to blood than from the CSF to blood and/or influx transporters from blood to CSF, both of which might contribute to a higher VGB concentration in CSF than in ECF [20]. In another study of antiepilepsy drugs (not VGB), CSF concentrations were 2.5–4.0 times higher than in ECF, determined by Rambeck *et al*. [21]. They suggested that this was due to overexpression of multidrug transporters, such as P-glycoprotein, in epileptic brain regions, although they did not compare drug concentrations in non-epileptic brain regions. Higher drug concentrations in CSF compared with microdialysate might also stem from CSF acting as a ‘sump’, into which the content of the extracellular fluid drains [31].

Two studies of VGB in the CSF of epilepsy patients by Halonen *et al*. [19] and by Pitkanen *et al*. [32] on chronic treatment reported similar VGB levels to those of the brain microdialysates of the TBI patients in the present study. After 3 months on 3 g VGB day^−1^, Halonen *et al*. found that the concentration of VGB in CSF was ∼4 μmol l^−1^, and on 1.5 g VGB day^−1^ the concentration was 2.4 μmol l^−1^ [19]. These values are comparable to VGB concentrations in microdialysates from TBI patients in the present study after multiple doses (*C*_av,br3_ = 4.2 μmol l^−1^, IQR 3.6–5.3 μmol l^−1^). Pitkanen *et al*. found that the VGB concentration in CSF after 7 months of treatment on an average of 2.7 g VGB day^−1^ was 2.8 μmol l^−1^ [32].

### Targets of vigabatrin

Although GABA is a known downstream target of VGB (see Introduction), in interpreting the results it needs to be acknowledged that brain injury itself may affect extracellular GABA levels. The fact that we did not see a very wide range of extracellular GABA concentrations across all VGB and control patients, even though there was presumably a varying degree of cell damage between patients, suggests that the extracellular GABA concentration was still tightly regulated and that any excess released was mostly mopped up by the cells. One of the patients (Patient 10), with two microdialysis catheters (A and B, respectively closer to and distant from a focal lesion), had a higher GABA concentration at A than at B, reflecting a higher VGB concentration at site A compared with B (Figure 3). However, because catheter A was closer to a focal lesion we cannot deduce whether the higher concentration of GABA at site A was due to more cellular damage or to higher VGB levels. A dedicated new study focusing on paired catheter comparisons within patients would be needed to shed more light on the issue of cell damage *vs.* extracellular GABA.

The literature indicates that GABA is released at high concentrations in situations where high concentrations of the excitatory amino acid glutamate are released [33], which has also been shown in human brain microdialysates (without VGB) during extreme conditions (ischaemia) [16]. In the present study, the patients' extracellular glutamate levels (Figure 5) were not high when gauged against the extreme values in the latter study [16], and also not high in the context of the range of values seen in a much larger microdialysis monitoring study in 223 TBI patients receiving neurocritical care, without VGB [4]. Thus, in the present study, the low extracellular GABA levels are consistent with the fairly low extracellular glutamate here, suggesting that the cells are not damaged enough to release much GABA.

The present study has helped to answer the following two important questions: (i) is VGB present at the target site; and (ii) is VGB exerting a response from the target receptor? Regarding point (i), the fact that an increase in VGB could be detected in the brain ECF of eight of the nine patients shows that VGB crosses the BBB. The reasons for inter- and intrapatient variability include general or local differences in BBB integrity, abundance and efficiency of efflux transporters and efficiency of uptake into cells. Regarding point (ii), VGB produces its pharmacological effect by binding to the GABA-T enzyme that is present in an intracellular location in glial and neuronal cells. Vigabatrin is efficiently taken up into neurons from the ECF by a high-affinity transporter [34] and has been shown to increase total brain GABA concentration, by *in vivo* magnetic resonance spectroscopy in patients [35–37]. Given that the intracellular concentration of GABA (1–6 mmol l^−1^) is much higher than the extracellular concentration (0.1–0.8 μmol l^−1^) [38], the magnetic resonance spectroscopy findings reflect that VGB increases intracellular GABA concentrations. However, a proportional relationship between intracellular and extracellular GABA concentrations has been suggested [39]. The brain extracellular GABA concentration can only be studied using microdialysis. The design of the present study anticipated that an increase in intracellular GABA (though not measurable by microdialysis), brought about by the action of VGB, might be detectable as an increase in extracellular GABA in the microdialysates. The results here suggest that the extracellular GABA concentration can also be influenced by VGB. Average GABA concentrations for four VGB patients increased with time, relative to the pre-dose baseline concentration, more so than in non-VGB control patients (Figure 4A), although the increases for each inter-dose period were not significant (*P* > 0.05). For Patient 10 (catheter A), microdialysate VGB concentrations rose with each dose and fell, with an overall ascending pattern across three doses, while GABA showed a somewhat similar but less accentuated pattern (Figure 3). There was little difference between the VGB patients and control patients in terms of absolute concentrations of GABA. However, there was a correlation between GABA and VGB concentrations in the VGB patients (*P* < 0.0001; Figure 4C).

Several studies have measured an increase in GABA concentration in CSF after VGB administration [11,17–19,24,30]. As CSF is a different compartment of the brain to ECF, these CSF results are not directly informative about ECF concentrations. In one such study, the average free GABA concentration in CSF of 11 epilepsy patients increased slightly from ∼125 to 150 nmol l^−1^ within 6 h of administration of a single VGB dose (50 mg kg^−1^, average 3.35 g) and continued to increase up to ∼175 and 200 nmol l^−1^ after 24 and 72 h, respectively [30]. The increase in GABA thus outlasted the presence of VGB by several days (because VGB fell to almost zero by 24 h), consistent with the primary mechanism of action of VGB, which is irreversible inhibition of the GABA-T enzyme. The brain microdialysates from TBI patients in the present study showed a trend for increase in GABA concentration from 0.27 to 0.51 μmol l^−1^ in 24 h, after two VGB doses in four patients (*P* = 0.06). Larger increases were seen after further multiple dosing [14]. Other studies have reported an increase in concentration of GABA in CSF after chronic VGB treatment. For example, in epilepsy patients, after 3 months of treatment with 3 g VGB day^−1^, the GABA concentration in CSF had increased from ∼0.12 to 0.25 μmol l^−1^ [19].

In a microdialysis PK study of VGB in rats, extracellular GABA concentration increased in the frontal cortex, but not in the hippocampus [28]. A lower VGB concentration was found in the ECF of the hippocampus, leading the authors to suggest that VGB did not reach a threshold concentration required for GABA increase. The increase in GABA concentration in the frontal cortex was delayed, beginning to increase 2 h after the peak VGB concentration in this part of the brain and continuing to increase as the VGB concentration was exponentially decreasing.

Future studies using microdialysis might investigate a conjugated form of extracellular GABA, homocarnosine (a GABA–histidine dipeptide), reported to increase from 3 to 12 μmol l^−1^ in CSF after chronic VGB treatment in epilepsy patients [11]. Homocarnosine was not measured in the present study owing to the lack of a reference standard.

### Limitations of the study

Vigabatrin concentration–effect relationships were outside the scope of our study; a fixed VGB dose was used because this was a small study, which was also too small to assess whether there was any effect on clinical outcome. An inherent limitation of microdialysis is that it is a focal technique and cannot give a global picture of VGB uptake (and downstream effects) throughout the brain, which is, of course, heterogeneous, both within a patient and between patients. Moreover, the focal nature of microdialysis means that VGB local extracellular concentrations might not necessarily correspond to VGB influences on clinical outcome. Also, the present study was in TBI patients, and it is unknown whether the extracellular concentration results would generalize to other patient populations, such as epilepsy sufferers.

In this study, we determined relative recovery of VGB only *in vitro*, rather than *in vivo*. While various approaches to measuring *in vivo* relative recovery exist [40], they would have been difficult to implement for practical and clinical reasons in these patients. Therefore, we also did not explore whether there were any changes in relative recovery of VGB with time *in vivo*. A potential source of variation in recovery with time might be a local inflammatory reaction induced by the microdialysis catheter. We did not explore inflammatory changes in the present study because they were outside its scope. However, in a dedicated study of cytokines, Helmy *et al*. found that time from injury was more significant than time from catheter insertion and, moreover, some of the cytokines showed little variation with time, suggesting that catheter *in vivo* recovery performance was maintained [41].

In this study, we did not distinguish between VGB enantiomers. The form of VGB that we used (Sabril) is a racemate consisting of a 1:1 mixture of *R*(−) and *S*(+) enantiomers of γ-vinyl-γ-aminobutyric acid, with the *S*(+) isomer being the pharmacologically active form [25]. Plasma *C*_max_ values for the *R*(−) enantiomer were 1.6- to 2-fold higher than for the *S*(+) enantiomer, while other plasma pharmacokinetics were very similar, reported in healthy adult volunteers and epileptic children [25,42]. The issue of enantiomers is potentially important in the context of penetration of VGB across the BBB, but we are unaware of literature reports. In TBI patients, a leaky BBB might be expected not to discriminate between *R*(−) and *S*(+) enantiomers *per se*, although the presumably greater plasma levels of the *R*(−) enantiomer might promote ensuing brain ECF concentrations. If transporter-mediated passage across the BBB were operating, it might be expected to favour the active *S*(+) enantiomer. The transport of VGB into neurons *in vitro* via an unknown high-affinity transporter is stereoselective for the *S*(+) enantiomer [34]. However, analysis to distinguish between the enantiomers was outside the scope of the present study.

Another limitation in this study was the presence of a small, unknown background peak at the same HPLC retention time as VGB. This unknown was present in the microdialysates from control patients, as well as prior to the start of dosing in microdialysates from the VGB patients (Supporting Information Table S3). In the control patients, this unknown increased only slightly with time, without statistical significance (Figure 4B). Therefore, in the VGB patients we regarded the increase in the VGB (plus unknown) peak on HPLC to be due predominantly to an increase in VGB. In future, more specific methods, such as liquid chromatography–tandem mass spectrometry, if available, might be advantageous to exclude any co-eluting unknown species from the VGB measurements.

This study highlights the challenges of performing a drug research study in a neurocritical care setting. Patients are severely injured, and sampling of blood plasma and brain microdialysates at the desirable time points for PK measurements are naturally subordinate to the clinical needs of the patient, which are paramount. Increases in VGB and GABA occur in a milieu of high concentrations of many other amino acids that complicate the chromatogram and limit accurate quantification of VGB and GABA at low concentrations. More specific methods, such as liquid chromatography–tandem mass spectrometry, may be advantageous in future studies to help overcome the difficulties of dealing with complex mixtures. A further limitation is that microdialysis samples only extracellular molecules and may underestimate any increases in the target GABA (which is mainly intracellular) as a result of VGB administration.

Clinical microdialysis studies of drugs in a neurocritical care setting may, in future, benefit from newer data-analysis methods, such as population-based PK modelling with sparse data sampling. This statistical methodology enables ‘sparse’ data from multiple patients to be combined to build up an integrated pharmacokinetic–pharmacodynamic model, with identification of covariates that can be used for future individualization of therapy [43–45]. Such approaches have proved advantageous in drug studies in other ‘vulnerable patient’ populations, such as paediatric patients [44]. Future clinical pharmacokinetic–pharmacodynamic drug trials in TBI patients could be planned with this type of PK analysis in mind. However, retrospective application to our present results would be difficult because of our fixed dosing and sampling regimen.

### Conclusions

Vigabatrin, given enterally to TBI patients, was detectable in plasma and brain microdialysates, indicating passage across the BBB, and enabled PK calculations. The increase in microdialysate VGB was followed by a modest increase in microdialysate GABA, albeit at very low extracellular levels, and was not seen in all patients. Vigabatrin and GABA increases were greater in abnormal regions of brain (as defined by computed tomography), as opposed to sites distant from focal lesions. Thus, more of the drug may reach damaged parts of the brain, which is potentially where the drug can have the most beneficial effect [14]. Increases in the extracellular concentration of VGB and GABA were more pronounced after repeated VGB doses than after a single dose. The continuous nature of microdialysis sampling of molecules from the brain ECF, which is the compartment to which glia and neurons are directly exposed, is an advantage over conventional CSF sampling employed in most studies of drugs in the brain.

The present study highlights important considerations for planning future drug PK studies involving cerebral microdialysis. These considerations include ensuring adequate intervals of sampling after doses for measuring rate constants, both in plasma and in the brain ECF, particularly as both uptake and elimination may be slow in the latter.

Many putative therapeutic drugs for TBI that seemed promising in animal models have failed in clinical trials. Recently, certain improvements in PK modelling are enabling better extrapolation from pre-clinical studies to man [46]. Moreover, new statistical methods, such as population PK with sparse data sampling, may be applied usefully to clinical drug studies using microdialysis in future (see previous subsection). Cerebral microdialysis data from key clinical studies, such as the present one in TBI patients, may enable refinement of such PK models, with the prospect of better translational research, more informed design of clinical trials, and individualization of therapy for better efficacy.

The cerebral microdialysis technique is suitable for application to drug molecules <100 kDa that are sufficiently hydrophilic to cross the microdialysis membrane. Microdialysis has the potential to assist in the development of putative neuroprotective agents by determining both the penetration into the brain extracellular fluid and the influence on downstream targets.

## Competing Interests

All authors have completed the Unified Competing Interest form at http://www.icmje.org/coi_disclosure.pdf (available on request from the corresponding author) and declare: (i) no support from any organization for the submitted work; (ii) PJH has been a Director of Technicam in the previous 3 years and has a patent cranial access device issued; (iii) no other relationships or activities that could appear to have influenced the submitted work.

The authors gratefully acknowledge study support from the Medical Research Council (grant numbers G9439390 ID 65883 and G0600986 ID 79068) and the Academy of Medical Sciences/Health Foundation, and support for authors as follows: RJS, National Institute for Health Research Flexibility and Sustainability Fund; KLHC, National Institute for Health Research Biomedical Research Centre, Cambridge (Neuroscience Theme; Brain Injury and Repair Theme) and the Medical Research Council (Acute Brain Injury Programme Grant); IT, Codman Inc. grant, The Evelyn Trust grant, MRC RESCUEicp trial grant and BP-TNK Kapitza Scholarship; JN, British Journal of Anaesthesia/Royal College of Anaesthetists Fellowship; and PJH, National Institute for Health Research Professorship, Academy of Medical Sciences/Health Foundation Senior Surgical Scientist Fellowship and the National Institute for Health Research Biomedical Research Centre, Cambridge (Neuroscience Theme; Brain Injury and Repair Theme). The authors thank Professors J. D. Pickard and M. Czosnyka for helpful comments with the manuscript. The authors also thank Mr I. Jalloh for assistance with patient demography and evaluation of computed tomography scans, and Ms L. Maskell for technical assistance with microdialysis and patient demography.

## Supporting Information

Figure S1 Bivariate scattergram of maximum vigabatrin (VGB) concentration (*C*_max,br1_) in brain microdialysates plotted *vs*. maximum VGB concentration in plasma (*C*_max,pl_), for the first VGB dose. Concentrations are expressed as μmol l^−1^. Patient identity numbers and, in the cases of patients with two catheters, catheter identities (A, B) are indicated for each data point

Figure S2 Average amino acid concentrations of serine, threonine, tyrosine and leucine measured in brain microdialysates for the 12 h period after the first dose, expressed as μmol l^−1^

Table S1 Demography for patients who received vigabatrin (VGB) and control patients

Table S2 Individual plasma and brain microdialysate vigabatrin concentrations (median, interquartile range) for first dose

Table S3 Individual vigabatrin concentrations (mean, interquartile range) in brain microdialysates after the first and third doses

## References

[1] Janowitz T, Menon DK (2010). Exploring new routes for neuroprotective drug development in traumatic brain injury. Sci Transl Med.

[2] Marklund N, Hillered L (2011). Animal modelling of traumatic brain injury in preclinical drug development: where do we go from here?. Br J Pharmacol.

[3] Hutchinson PJ, O'Connell MT, Al-Rawi PG, Maskell LB, Kett-White R, Gupta AK, Richards HK, Hutchinson DB, Kirkpatrick PJ, Pickard JD (2000). Clinical cerebral microdialysis: a methodological study. J Neurosurg.

[4] Timofeev I, Carpenter KL, Nortje J, Al-Rawi PG, O'Connell MT, Czosnyka M, Smielewski P, Pickard JD, Menon DK, Kirkpatrick PJ, Gupta AK, Hutchinson PJ (2011). Cerebral extracellular chemistry and outcome following traumatic brain injury: a microdialysis study of 223 patients. Brain.

[5] Dahyot-Fizelier C, Timofeev I, Marchand S, Hutchinson P, Debaene B, Menon D, Mimoz O, Gupta A, Couet W (2010). Brain microdialysis study of meropenem in two patients with acute brain injury. Antimicrob Agents Chemother.

[6] Dahyot-Fizelier C, Frasca D, Gregoire N, Adier C, Mimoz O, Debaene B, Couet W, Marchand S (2013). Microdialysis study of cefotaxime cerebral distribution in patients with acute brain injury. Antimicrob Agents Chemother.

[7] Lippert B, Metcalf BW, Jung MJ, Casara P (1977). 4-amino-hex-5-enoic acid, a selective catalytic inhibitor of 4-aminobutyric-acid aminotransferase in mammalian brain. Eur J Biochem.

[8] Palfreyman MG, Schechter PJ, Buckett WR, Tell GP, Koch-Weser J (1981). The pharmacology of GABA-transaminase inhibitors. Biochem Pharmacol.

[9] Schechter PJ, Hanke NF, Grove J, Huebert N, Sjoerdsma A (1984). Biochemical and clinical effects of gamma-vinyl GABA in patients with epilepsy. Neurology.

[10] Ben-Menachem E (1989). Pharmacokinetic effects of vigabatrin on cerebrospinal fluid amino acids in humans. Epilepsia.

[11] Pitkanen A, Matilainen R, Ruutiainen T, Riekkinen P (1988). Levels of total gamma-aminobutyric acid (GABA), free GABA and homocarnosine in cerebrospinal fluid of epileptic patients before and during gamma-vinyl-GABA (vigabatrin) treatment. J Neurol Sci.

[12] Lindberger M, Luhr O, Johannessen SI, Larsson S, Tomson T (2003). Serum concentrations and effects of gabapentin and vigabatrin: observations from a dose titration study. Ther Drug Monit.

[13] Rey E, Pons G, Olive G (1992). Vigabatrin. Clinical pharmacokinetics. Clin Pharmacokinet.

[14] Carpenter KL, Timofeev I, Nortje J, Czosnyka M, Pickard JD, Hutchinson PJ (2012). A microdialysis study of oral vigabatrin administration in head injury patients: preliminary evaluation of multimodality monitoring. Acta Neurochir Suppl.

[15] Gratzfeld-Huesgen A (1999).

[16] Hutchinson PJ, O'Connell MT, Al-Rawi PG, Kett-White CR, Gupta AK, Maskell LB, Pickard JD, Kirkpatrick PJ (2002). Increases in GABA concentrations during cerebral ischaemia: a microdialysis study of extracellular amino acids. J Neurol Neurosurg Psychiatry.

[17] Ben-Menachem E, Persson LI, Schechter PJ, Haegele KD, Huebert N, Hardenberg J, Dahlgren L, Mumford JP (1989). The effect of different vigabatrin treatment regimens on CSF biochemistry and seizure control in epileptic patients. Br J Clin Pharmacol.

[18] Riekkinen PJ, Ylinen A, Halonen T, Sivenius J, Pitkanen A (1989). Cerebrospinal fluid GABA and seizure control with vigabatrin. Br J Clin Pharmacol.

[19] Halonen T, Lehtinen M, Pitkanen A, Ylinen A, Riekkinen PJ (1988). Inhibitory and excitatory amino acids in CSF of patients suffering from complex partial seizures during chronic treatment with gamma-vinyl GABA (vigabatrin). Epilepsy Res.

[20] Zhuang Y, Fraga CH, Hubbard KE, Hagedorn N, Panetta JC, Waters CM, Stewart CF (2006). Topotecan central nervous system penetration is altered by a tyrosine kinase inhibitor. Cancer Res.

[21] Rambeck B, Jurgens UH, May TW, Pannek HW, Behne F, Ebner A, Gorji A, Straub H, Speckmann EJ, Pohlmann-Eden B, Loscher W (2006). Comparison of brain extracellular fluid, brain tissue, cerebrospinal fluid, and serum concentrations of antiepileptic drugs measured intraoperatively in patients with intractable epilepsy. Epilepsia.

[22] Hoke JF, Yuh L, Antony KK, Okerholm RA, Elberfeld JM, Sussman NM (1993). Pharmacokinetics of vigabatrin following single and multiple oral doses in normal volunteers. J Clin Pharmacol.

[23] Durham SL, Hoke JF, Chen TM (1993). Pharmacokinetics and metabolism of vigabatrin following a single oral dose of [14C]vigabatrin in healthy male volunteers. Drug Metab Dispos.

[24] Schechter PJ (1989). Clinical pharmacology of vigabatrin. Br J Clin Pharmacol.

[25] Haegele KD, Schechter PJ (1986). Kinetics of the enantiomers of vigabatrin after an oral dose of the racemate or the active S-enantiomer. Clin Pharmacol Ther.

[26] Weiss J, Kerpen CJ, Lindenmaier H, Dormann SM, Haefeli WE (2003). Interaction of antiepileptic drugs with human P-glycoprotein in vitro. J Pharmacol Exp Ther.

[27] Crowe A, Teoh YK (2006). Limited P-glycoprotein mediated efflux for anti-epileptic drugs. J Drug Target.

[28] Tong X, Ratnaraj N, Patsalos PN (2009). Vigabatrin extracellular pharmacokinetics and concurrent gamma-aminobutyric acid neurotransmitter effects in rat frontal cortex and hippocampus using microdialysis. Epilepsia.

[29] Bouw MR, Gardmark M, Hammarlund-Udenaes M (2000). Pharmacokinetic-pharmacodynamic modelling of morphine transport across the blood-brain barrier as a cause of the antinociceptive effect delay in rats – a microdialysis study. Pharm Res.

[30] Menachem EB, Persson LI, Schechter PJ, Haegele KD, Huebert N, Hardenberg J, Dahlgren L, Mumford JP (1988). Effects of single doses of vigabatrin on CSF concentrations of GABA, homocarnosine, homovanillic acid and 5-hydroxyindoleacetic acid in patients with complex partial epilepsy. Epilepsy Res.

[31] Helmy A, De Simoni MG, Guilfoyle MR, Carpenter KL, Hutchinson PJ (2011). Cytokines and innate inflammation in the pathogenesis of human traumatic brain injury. Prog Neurobiol.

[32] Pitkanen A, Matilainen R, Ruutiainen T, Lehtinen M, Riekkinen P (1988). Effect of vigabatrin (gamma-vinyl GABA) on amino acid levels in CSF of epileptic patients. J Neurol Neurosurg Psychiatry.

[33] Saransaari P, Oja SS (1998). Release of endogenous glutamate, aspartate, GABA, and taurine from hippocampal slices from adult and developing mice under cell-damaging conditions. Neurochem Res.

[34] Schousboe A, Larsson OM, Seiler N (1986). Stereoselective uptake of the GABA-transaminase inhibitors gamma-vinyl GABA and gamma-acetylenic GABA into neurons and astrocytes. Neurochem Res.

[35] Petroff OA, Rothman DL, Behar KL, Collins TL, Mattson RH (1996). Human brain GABA levels rise rapidly after initiation of vigabatrin therapy. Neurology.

[36] Petroff OA, Behar KL, Mattson RH, Rothman DL (1996). Human brain gamma-aminobutyric acid levels and seizure control following initiation of vigabatrin therapy. J Neurochem.

[37] Petroff OA, Rothman DL, Behar KL, Mattson RH (1996). Human brain GABA levels rise after initiation of vigabatrin therapy but fail to rise further with increasing dose. Neurology.

[38] Wu Y, Wang W, Diez-Sampedro A, Richerson GB (2007). Nonvesicular inhibitory neurotransmission via reversal of the GABA transporter GAT-1. Neuron.

[39] Petroff OA, Rothman DL (1998). Measuring human brain GABA in vivo: effects of GABA-transaminase inhibition with vigabatrin. Mol Neurobiol.

[40] Shannon RJ, Carpenter KL, Guilfoyle MR, Helmy A, Hutchinson PJ (2013). Cerebral microdialysis in clinical studies of drugs: pharmacokinetic applications. J Pharmacokinet Pharmacodyn.

[41] Helmy A, Carpenter KL, Menon DK, Pickard JD, Hutchinson PJ (2011). The cytokine response to human traumatic brain injury: temporal profiles and evidence for cerebral parenchymal production. J Cereb Blood Flow Metab.

[42] Rey E, Pons G, Richard MO, Vauzelle F, D'Athis P, Chiron C, Dulac O, Beaumont D, Olive G (1990). Pharmacokinetics of the individual enantiomers of vigabatrin (gamma-vinyl GABA) in epileptic children. Br J Clin Pharmacol.

[43] Schaeftlein A, Minichmayr IK, Kloft C (2014). Population pharmacokinetics meets microdialysis: benefits, pitfalls and necessities of new analysis approaches for human microdialysis data. Eur J Pharm Sci.

[44] Knibbe CA, Danhof M (2011). Individualized dosing regimens in children based on population PKPD modelling: are we ready for it?. Int J Pharm.

[45] Duffull SB, Wright DF, Winter HR (2011). Interpreting population pharmacokinetic-pharmacodynamic analyses – a clinical viewpoint. Br J Clin Pharmacol.

[46] de Lange EC (2013). The mastermind approach to CNS drug therapy: translational prediction of human brain distribution, target site kinetics, and therapeutic effects. Fluids Barriers CNS.

