# Supplementary figures and images for: Monitoring vigabatrin in head injury patients by cerebral microdialysis: obtaining pharmacokinetic measurements in a neurocritical care setting
Source: Br J Clin Pharmacol. 2014 Oct 20;78(5):981–95. doi: 10.1111/bcp.12414 (PMC4243872; doi:10.1111/bcp.12414)

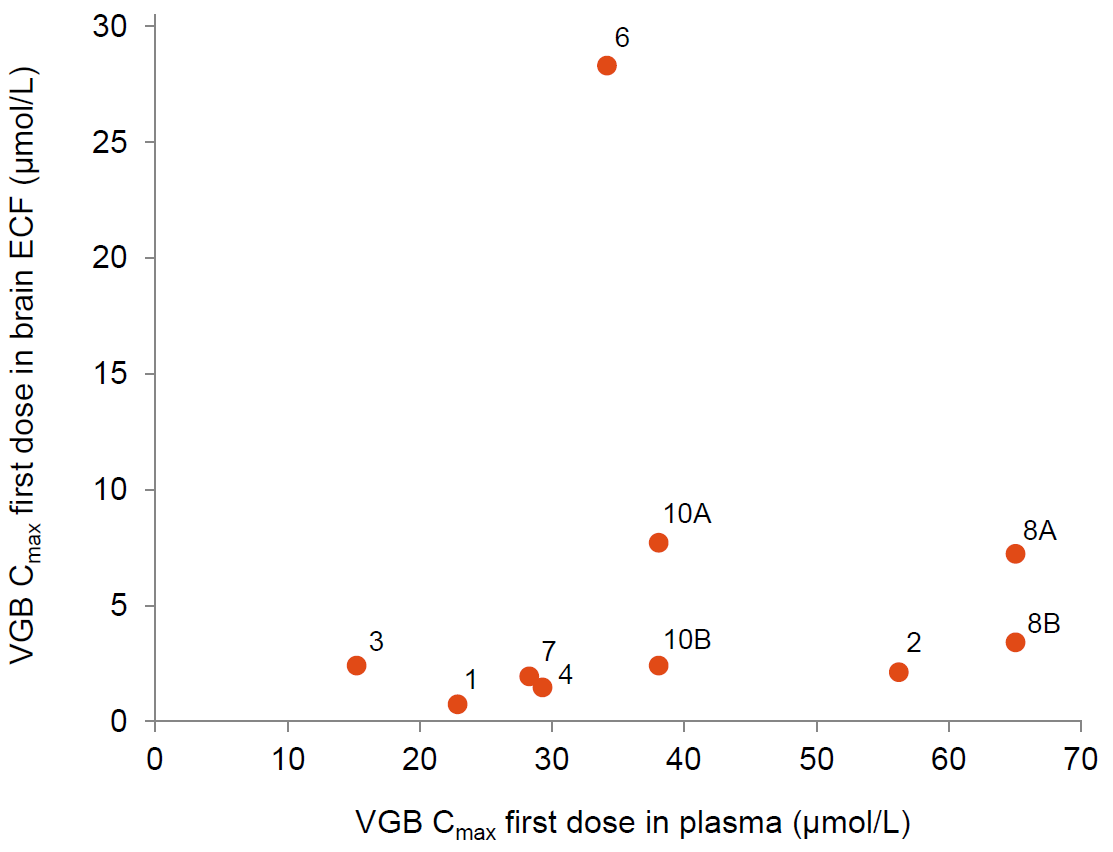

Supplement: Figure S1 — Bivariate scattergram of maximum vigabatrin (VGB) concentration (Cmax,br1) in brain microdialysates plotted vs. maximum VGB concentration in plasma (Cmax,pl), for the first VGB dose. Concentrations are expressed as μmol l−1. Patient identity numbers and, in the cases of patients with two catheters, catheter identities (A, B) are indicated for each data point [file bcp0078-0981-sd1.tif]

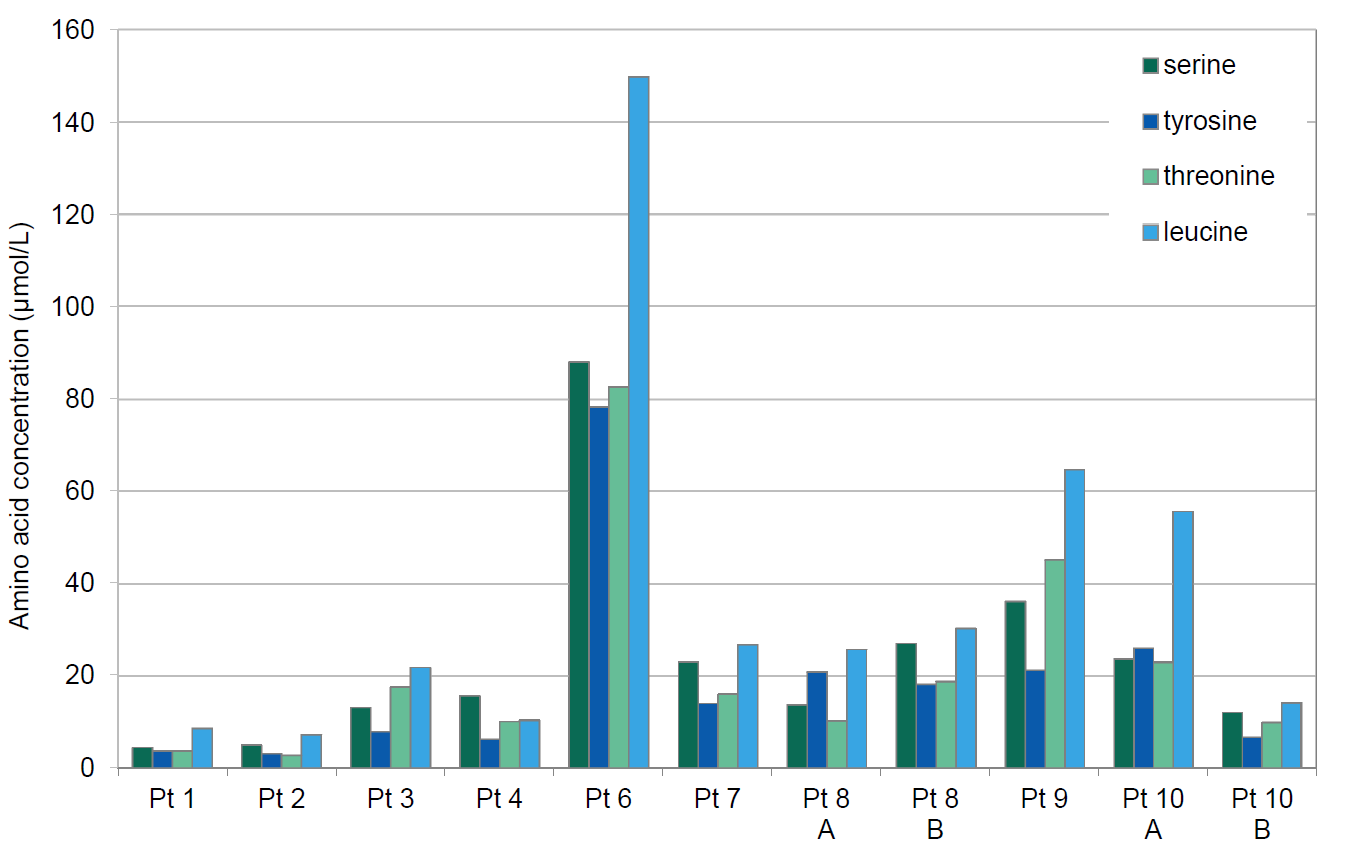

Supplement: Figure S2 — Average amino acid concentrations of serine, threonine, tyrosine and leucine measured in brain microdialysates for the 12 h period after the first dose, expressed as μmol l−1 [file bcp0078-0981-sd2.tif]
