# Supplementary material for: Monitoring vigabatrin in head injury patients by cerebral microdialysis: obtaining pharmacokinetic measurements in a neurocritical care setting
Source: Br J Clin Pharmacol. 2014 Oct 20;78(5):981–95. doi: 10.1111/bcp.12414 (PMC4243872; doi:10.1111/bcp.12414)
Supplement: Table S1 — Demography for patients who received vigabatrin (VGB) and control patients [file bcp0078-0981-sd3.docx]

**Supplementary Table 1: Demography for patients who received vigabatrin (VGB) and control patients.**

| **VGB patient i.d. no.** | **Age (y)** | **Sex** | **GCS** | **Main finding on CT scan** | **Injury Marshall Grade (CT)** | **Craniect-omy** | **Position of catheter** | **Microdialysis start post-injury day** | **First dose post-injury day** |
| --- | --- | --- | --- | --- | --- | --- | --- | --- | --- |
| 1 | 33 | M | 6 | -- | -- | No | RF | 1 | 3 |
| 2 | 21 | F | 3 | contusions, tSAH | 2d | Yes | RF | 2 | 3 |
| 3 | 16 | M | 8 | -- | -- | Yes | RF | 2 | 4 |
| 4 | 42 | M | 14 | contusions, ASDH, tSAH | 6d | Yes | LF | 2 | 3 |
| 5*** | 38 | M | 3 | no traumatic haemorrhage | 1/2a | No | RF | 2 | -- |
| 6 | 66 | M | 7 | residual ASDH, tSAH, IVH | 5b | Yes | RF | 2 | 3 |
| 7 | 17 | M | 3 | tSAH | 2d | No | RF | 3 | 4 |
| 8 | 58 | F | 8 | ASDH, contusions, tSAH | 4 | Yes | A: Left CTO  B: LF | 1 | 3 |
| 9 | 33 | M | 14 | contusions, tSAH | 2c | No | RF | 1 | 6 |
| 10 | 35 | M | 4 | ASDH, contusions, tSAH | 6b | Yes | A: RF CTO  B: LF | 1 | 3 |
| **Control**  **patient i.d. no.** | **Age**  **(y)** | **Sex** | **GCS** | **Main finding on CT scan** | **Injury Marshall Grade (CT)** | **Craniect-omy** | **Position of catheter** | **Microdialysis start post-injury day** | **Start of control monitoring post-injury day** |
| 11 | 50 | M | 3 | ASDH, tSAH, contusions | 2d | No | RF | 1 | 3 |
| 12 | 33 | M | 5 | -- | -- | No | -- | 1 | 2 |
| 13 | 56 | M | 3 | tSAH, ASDH, contusions | 4 | Yes | RF | 3 | 7 |
| 14 | 24 | M | 4 | tSAH, contusions | 3 | No | RF | 1 | 3 |
| 15 | 17 | F | -- | ASDH, contusions, tSAH | 6d | Yes | A: RF  B: LF CTO | A: 1  B: 2 | A: 4  B: 4 |
| 16 | 39 | M | 7 | tSAH, IVH contusions | 2d | No | RF | 2 | 4 |
| 17 | 49 | F | -- | contusions, tSAH | 2d | No | RF | 0 | 2 |

*Abbreviations:* GCS, Glasgow Coma Scale score on admission; tSAH, traumatic subarachnoid haemorrhage; RF, right frontal; LF, left frontal; ASDH, acute subdural haemorrhage; IVH, intraventricular haemorrhage; CTO, craniectomy site. Blank entries (--) indicate no data.

*Notes: ** Data from Patient 5 was excluded from results due to uncertain timing of doses.
