# Supplementary material for: Monitoring vigabatrin in head injury patients by cerebral microdialysis: obtaining pharmacokinetic measurements in a neurocritical care setting
Source: Br J Clin Pharmacol. 2014 Oct 20;78(5):981–95. doi: 10.1111/bcp.12414 (PMC4243872; doi:10.1111/bcp.12414)
Supplement: Table S2 — Individual plasma and brain microdialysate vigabatrin concentrations (median, interquartile range) for first dose [file bcp0078-0981-sd4.docx]

**Supplementary Table 2: Individual plasma and brain microdialysate vigabatrin concentrations (median, IQR) for 1^st^ dose.**

|  | | **Plasma vigabatrin pharmacokinetics** | | | **Brain vigabatrin pharmacokinetics (microdialysates)** | | | |
| --- | --- | --- | --- | --- | --- | --- | --- | --- |
| **Patient i.d. no.** | | **C_max,pl_ (µM)** | **T_max,pl_ (h)** | **AUC_0-t,pl_**  **(h·µM)** | **C_max,br1_ (µM)** | **T_max,br1_ (h)** | **AUC_0-t,br1_**  **(h·µM)** | **C_max (br/pl)_ ratio** |
| 1 | | 22.9 | 4 | 218.4 | 0.74 | 3.3 | 2.22 | 0.03 |
| 2 | | 56.2 | 4 | 410.8 | 2.12 | 3.8 | 20.3 | 0.04 |
| 3 | | 15.2 | 4 | 80.9 | 2.40 | 9.5 | 19.5 | 0.16 |
| 4 | | 29.3 | 4 | 286.2 | 1.47 | 9.8 | 14.6 | 0.05 |
| 6 | | 34.2 | 2 | 240.9 | 28.3 | 3.2 | 181 | 0.83 |
| 7 | | 28.3 | 2 | 166.6 | 1.94 | 6.6 | 13.6 | 0.07 |
| 8 | A | 65.1 | 2 | 459.5 | 7.23 | 5.7 | 41.1 | 0.11 |
|  | B |  |  |  | 3.41 | 4.7 | 28.5 | 0.05 |
| 9 | | NS | NS | NS | 4.64 | 8.1 | 40.8 | ND |
| 10 | A | 38.1 | 2 | 257.3 | 7.71 | 5.1 | 67.5 | 0.20 |
|  | B |  |  |  | 2.41 | 5.1 | 23.2 | 0.06 |
| Median | | 31.7 | 3 | 249.1 | 2.41 | 5.1 | 23.2 | 0.07 |
| 25% Q | | 26.9 | 2 | 205.4 | 2.03 | 4.3 | 17.0 | 0.05 |
| 75% Q | | 42.6 | 4 | 317.3 | 5.94 | 7.4 | 41.0 | 0.15 |

*Abbreviations:* C_max,pl_ , maximum VGB concentration in plasma after 1^st^ dose; T_max,pl_ ,time-point with highest VGB concentration in plasma after 1^st^ dose; AUC_0‑t,pl_ , area under the plasma VGB concentration-time curve for 0 - 11.5 h after 1^st^ dose; C_max_,_br1_ , peak brain microdialysate VGB concentration after 1^st^ dose; T_max,br1_ , time-point with highest VGB concentration in brain microdialysates after 1^st^ dose; AUC_0-t,br1_ , area under brain microdialysate VGB concentration-time curve for 0 - 12 h after 1^st^ dose; C_max (br/pl)_ ratio, the ratio of maximum VGB concentration in brain microdialysates to that in plasma (C_max,br1_/C_max_,_pl_); NS, no sample; ND, not possible to determine as no plasma samples.
