# Supplementary material for: Monitoring vigabatrin in head injury patients by cerebral microdialysis: obtaining pharmacokinetic measurements in a neurocritical care setting
Source: Br J Clin Pharmacol. 2014 Oct 20;78(5):981–95. doi: 10.1111/bcp.12414 (PMC4243872; doi:10.1111/bcp.12414)
Supplement: Table S3 — Individual vigabatrin concentrations (mean, interquartile range) in brain microdialysates after the first and third doses [file bcp0078-0981-sd5.docx]

**Supplementary Table 3: Individual vigabatrin concentrations (mean, IQR) in brain microdialysates after the 1^st^ and 3^rd^ doses.**

|  | | **Pre-dose**  **background** | **1^st^ Dose** | | **3^rd^ Dose** | | **Accumulation ratio** |
| --- | --- | --- | --- | --- | --- | --- | --- |
| **Patient i.d. no.** | | **C_av,pre_ (µM)** | **C_max,br1_**  **(µM)** | **C_av,br1_**  **(µM)** | **C_max,br3_**  **(µM)** | **C_av,br3_ (µM)** | **R_ac_**  **(C_av,br3_/C_av,br1_)** |
| 1 | | 0.33 | 0.74 | 0.52 | 2.62 | 1.83 | 3.6 |
| 2 | | 1.94 | 2.12 | 1.69 | 4.69 | 4.42 | 2.6 |
| 3 | | 1.31 | 2.40 | 1.86 | -- | -- | -- |
| 4 | | 1.19 | 1.47 | 1.20 | 1.58 | 1.35 | 1.1 |
| 6 | | 0.00 | 28.3 | 23.1 | 72.9 | 56.7 | 2.5 |
| 7 | | 0.00 | 1.94 | 1.17 | 7.16 | 5.34 | 4.6 |
| 8 | A | 1.92 | 7.23 | 4.74 | 7.08 | 5.23 | 1.1 |
|  | B | 2.30 | 3.41 | 2.44 | 4.57 | 3.88 | 1.6 |
| 9 | | 2.58 | 4.65 | 3.59 | 4.13 | 3.53 | 1.0 |
| 10 | A | 0.89 | 7.71 | 5.55 | 12.7 | 9.12 | 1.6 |
|  | B | 0.95 | 2.41 | 1.90 | 5.75 | 3.91 | 2.1 |
| Median | | 1.19 | 2.41 | 1.90 | 5.22 | 4.16 | 1.8 |
| 25% Q | | 0.61 | 2.03 | 1.44 | 4.24 | 3.62 | 1.2 |
| 75% Q | | 1.93 | 5.94 | 4.16 | 7.14 | 5.31 | 2.6 |

*Abbreviations and footnotes:* C_av,pre_ , average background concentration before 1st VGB dose, over a mean (±SD) period of 4.2 ± 2.3 h. Background due to co-eluting unknown peak in the HPLC chromatogram, with same retention time as VGB and quantified as if it were VGB. C_av,br1_ , average microdialysate VGB concentration after 1^st^ dose; C_max,br3_ , peak microdialysate VGB concentration after 3^rd^ dose; C_av,br3_ , average microdialysate VGB concentration after 3^rd^ dose; R_ac_ , accumulation ratio calculated as C_av,br3_ divided by C_av,br1_ ; blank entry (--) indicates no data (Patient 3’s microdialysis discontinued after 2^nd^ dose). For other abbreviations see Supplementary Table 2.
